# Multi-level Model Product Lines: Open and Closed Variability for Modelling Language Families

**DOI:** 10.1007/978-3-030-45234-6_8

**Published:** 2020-03-13

**Authors:** Juan de Lara, Esther Guerra

**Affiliations:** 8 University of Paderborn, Paderborn, Germany; 9 ICREA, Open University of Catalonia, Barcelona, Spain; Universidad Autónoma de Madrid, Madrid, Spain

**Keywords:** Meta-modelling, Multi-level modelling, Product lines, Domain-specific languages, MetaDepth

## Abstract

Modelling is an essential activity in software engineering processes. It typically involves two meta-levels: one includes meta-models that describe modelling languages, and the other contains models built by instantiating those meta-models. *Multi-level modelling* generalizes this approach by allowing models to span an arbitrary number of meta-levels.

A scenario that profits from multi-level modelling is the definition of language families that become specialized by successive refinements at subsequent meta-levels, hence promoting language reuse. This enables an *open* set of variability options for the possible specializations of a given language. However, multi-level modelling lacks the ability to express *closed* variability regarding the supported language primitives and their realizations. This limits the reuse opportunities of a language family. To improve this situation, we propose a novel combination of product lines with multi-level modelling to cover both open and closed variability. Our proposal is backed by a formal theory that guarantees correctness, and is implemented atop the MetaDepth multi-level modelling tool.

## 1 Introduction

Modelling is intrinsic to most engineering disciplines. Within software engineering, it plays a pivotal role in model-driven engineering (MDE) [43]. This is a software construction paradigm where models are actively used to describe, analyse, validate, verify, generate code and maintain the application to be built, among other activities.

Models are built using modelling languages, which can be either general-purpose, like the UML [46], or domain-specific languages (DSLs) tailored to a specific concern [25]. In MDE, the abstract syntax of modelling languages is defined through a meta-model that describes the primitives that models can use one meta-level below. This modelling approach, which is the standard nowadays, constrains engineers to confine their models within one meta-level (the "model" level).

Some researchers have observed that domain modelling can benefit from the use of more than one meta-level [6, 14, 17, 19, 29]. This way of modelling – called multi-level modelling [4] or deep meta-modelling [12] – results in simpler models in scenarios that involve the type-object pattern [6, 14, 30]. Moreover, it permits defining language families (e.g., for process modelling), which can be specialized to specific domains (e.g., software process modelling, industrial process modelling) via instantiation

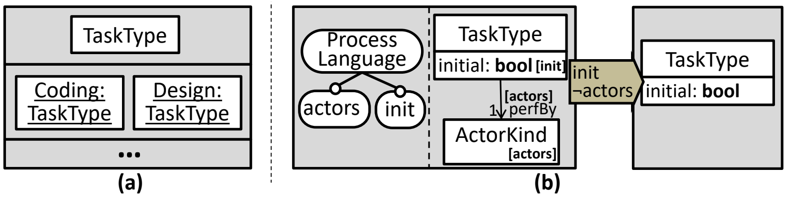

**Fig. 1.** (a) Open variability through instantiation. (b) Closed variability through product lines.

at lower meta-levels [15]. Instantiation is an *open* variability mechanism that permits the language customization by specializing the language primitives for a domain, or adding new ones via linguistic extensions [12]. Fig. 1(a) shows a tiny process modelling language that defines the primitive TaskType, which is customized by instantiation in the lower meta-level for the software process modelling domain (Coding and Design). However, multi-level modelling lacks support for expressing optionality of language primitives or alternative primitive realizations. This prevents wider language reuse and customization possibilities.

Software product lines (SPLs) encompass methods, tools and techniques to engineer collections of similar software systems using a common means of production [32, 35]. SPLs support *closed* variability, where a concrete software product is obtained by selecting among a finite set of available features (i.e., by setting a *configuration*). SPL techniques have been applied to language engineering to define product lines of languages representing a close set of predefined language variants [20, 34, 47]. As an example, Fig. 1(b) shows a process modelling language product line with two configurable features: actors and initial tasks. Selecting a configuration of features (in the figure, initial tasks but no actors) yields a language variant. Languages so defined can be configured with respect to the primitives they offer and their realization, but cannot be specialized for specific domains as this requires from open variability mechanisms.

To improve current language reuse techniques, we propose combining multi-level modelling and product lines. This allows the definition of highly configurable language families that profit from both open variability (as given by instantiation) and closed variability (as given by configuration). This way, this paper makes the following contributions: (i) a novel notion of multi-level model product line; (ii) a theory that guarantees the correctness of (certain) interleavings of instantiation and configuration steps; and (iii) an implementation of these ideas on top of the METADEPTH tool [12].

**Paper organization.** Section 2 introduces multi-level modelling and identifies the challenges tackled in this paper. Section 3 provides a light formalization of multi-level modelling, which is extended with product line techniques in Section 4. Section 5 describes tool support. Section 6 discusses related research, and Section 7 ends with the conclusions and future work. An appendix includes the proofs of the theorems in the paper.

## 2 Multi-level modelling: intuition and challenges

In this section, we introduce the main concepts of multi-level modelling by example (Section 2.1), and then discuss the challenges that we aim to tackle (Section 2.2).

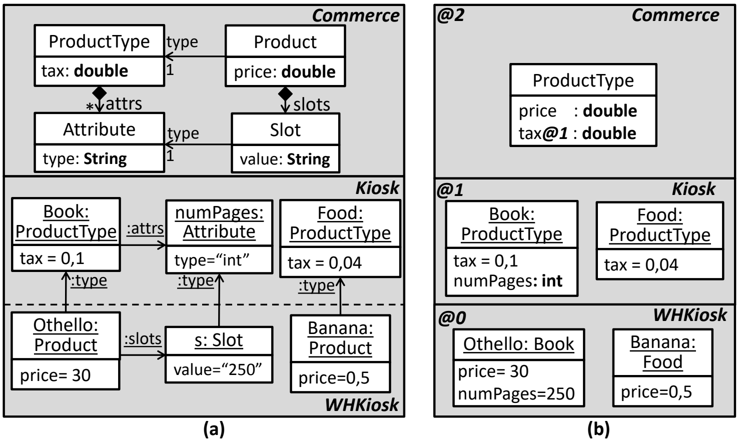

**Fig. 2.** Commerce example using (a) standard modelling and (b) multi-level modelling.

### 2.1 Multi-level modelling, by example

Multi-level modelling permits the definition of models using multiple meta-levels [6, 14]. To understand its rationale, assume we would like to create a language to define commerce information systems (a standard example often used in the multi-level modelling literature [6, 14]). This language should allow defining *product types* (like *books* or *food*) which have a *tax*, as well as *products* of the defined types (like *Othello* or *banana*) which have a *price*. Moreover, some product types may need to define specific properties, like the number of *pages* in books.

Fig. 2(a) shows a solution using two meta-levels. In this solution, the meta-model of the language uses the *type-object* pattern [30] to emulate the typing relation between Product and ProductType. In addition, classes Attribute and Slot permit defining properties in ProductTypes and assigning them a value in Products (called *dynamic features* pattern in [14]). The model in the bottom meta-level represents an information system for Kiosks, and defines the product types Book and Food. The model also defines the products sold by a particular kiosk: the Othello book and Bananas.

On reflection, one can realize that this solution emulates two meta-levels within one, as we convey with the dashed line in Fig. 2(a). Therefore, Fig. 2(b) shows an alternative multi-level solution using three meta-levels. The top level defines just ProductType, which is instantiated at the next level to create Book and Food product types, which in turn are instantiated at the bottom level to create specific products. Hence, elements in this approach are called *clabjects* [2] (from the contraction of the words *cla*ss and o*bject*), as they are types for the elements in the level below, and instances of the elements in the level above (see for instance Book).

The multi-level solution leads to a simpler model (with fewer elements) as it requires just a clabject to represent both ProductType and Product. However, one needs to control the properties of instances beyond the next meta-level. In the example, we need to control that the direct instances of ProductType have a tax, and the instances of

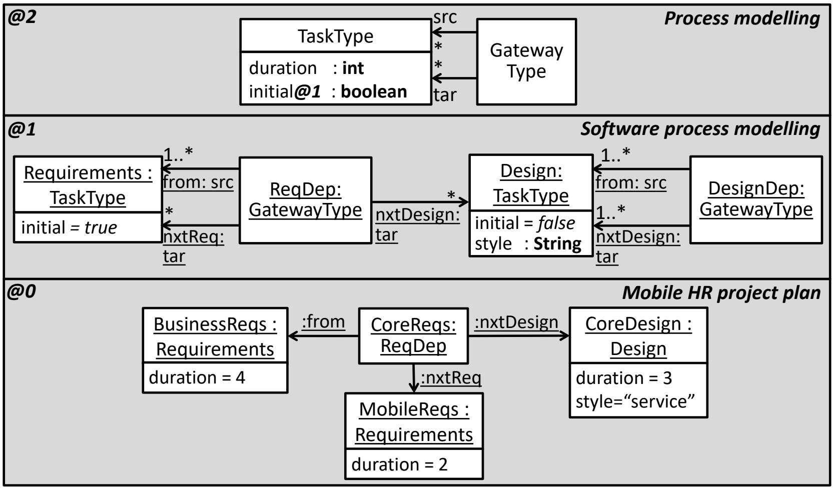

**Fig. 3.** Multi-level model for process modelling, and application to software process modelling.

its instances have a price. For this purpose, we use a *deep characterization* mechanism called potency [2, 4]. This is a natural number, or zero, which governs the instantiation depth of elements. Fig. 2(b) depicts the potency after the "@" symbol, and the elements that do not declare potency take the potency from their container (e.g., attribute price takes its potency from ProductType, and this from the Commerce model). When an element is instantiated, the instance gets the potency of the element minus 1. Elements with potency 0 are pure instances and cannot be instantiated. This way, attribute ProductType.tax is instantiated into Book.tax and Food.tax, which therefore have potency 0 and can receive values. As model Commerce has potency 2, it can be instantiated at the two subsequent meta-levels. The potency of a model is often called its *level* [6].

Sometimes, it is not possible to foresee every possible property required by clabject instances several meta-levels below, like the number of pages in books. To handle those cases, multi-level modelling supports *linguistic extensions*. These are clabjects or features with no ontological type, but with a linguistic type which corresponds to the meta-modelling primitive used to create it (see Orthogonal Classification Architecture in [5] for more details). As an example, Book.numPages is a linguistic extension modelling a property specific to Book but not to other product types. Instead, in the two-level solution in Fig. 2(a), the properties of specific ProductTypes need to be explicitly modelled by classes Attribute and Slot, leading to more complexity.

## 2.2 Improving reuse in multi-level modelling: some challenges

Multi-level modelling enables language reuse by supporting the definition of language families. For example, Fig. 3 shows at the top a generic process modelling language that can be used to define process modelling languages for different domains, like education, software engineering, or production engineering. The language is designed to consider three levels. Level 2 contains the language definition, consisting of primitives

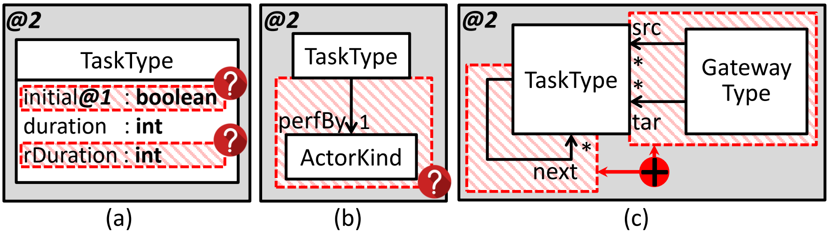

**Fig. 4.** Examples of variability needs: (a) optional attributes, (b) optional primitives, (c) alternative primitive realizations.

to define task and gateway types. Level 1 contains language specializations for specific domains. The figure shows the case for the software engineering domain, which defines the task types Requirements and Design, and two gateway types: ReqDep to transition from requirement tasks to either design or requirement tasks, and DesignDep to declare dependencies between design tasks. Finally, level 0 contains domain-specific processes. The one in the figure declares three tasks and one gateway.

This example shows how instantiation permits customizing the language primitives offered at the top level for particular domains, and how linguistic extensions (e.g., attribute Design.style at level 1 in Fig. 3) allow adding domain-specific primitives to language specializations. However, the following scenarios require further facilities that enable a better fit for particular domains and increase language reuse.

– **Alternative realizations**. A language primitive may be realised in different ways, each more adequate than the others depending on the domain. For example, in Fig. 3, dependencies between task types are modelled by GatewayType. However, in domains that do not require distinguishing gateway types, a simpler representation of dependencies as a reference between TaskTypes is enough (see Fig. 4(c)). Unfortunately, multi-level modelling does not support this kind of variability.
– **Primitive excess**. Some offered language primitives may be unnecessary in simple domains. This can be controlled by not instantiating the primitive, but still, withdrawing the needless primitives to simplify the language usage may be a better option. Moreover, there are problematic situations. First, if the primitive is an attribute (like initial in Fig. 4(a)), then it becomes instantiated by force, polluting the model with unnecessary information. Second, some mandatory primitives may not be needed in certain domains. For example, in Fig. 4(b), the language designer assumes that any TaskType (e.g., Requirements) will be performed by one ActorKind (e.g., Analyst or DomainExpert). However, there may be domains that do not involve actors (e.g., if tasks are automated), but the mandatory relation perfBy forces having instances of ActorKind associated to instances of TaskType.
– **Deferred variability resolution and exploratory modelling**. The decision about the inclusion or not of a primitive may not be clear when the language is instantiated for a domain, but this is determined later at lower meta-levels. For example, in Fig. 4(a), an engineer might hesitate whether, in addition to the expected task duration (attribute duration), s/he may want to store the real task duration (attribute rDuration with potency 2), in which case, s/he may prefer deferring the decision to levels 1 or 0. In general, resolving all variability in a language family at the top level may be hasty in some cases, as the suitability of a primitive may become evident only when a language has reached certain specificity (i.e., at lower meta-levels). Moreover, enabling modelling before resolving the variability may be good for exploratory purposes.

To tackle these challenges, we incorporate variability into multi-level models taking ideas from SPLs. As a first step, next we formalize multi-level models.

## 3 A formal foundation for multi-level modelling

We start defining the structure of models equipped with deep characterization, which we call *deep* models. We represent models at different meta-levels in a uniform way, in order to cope with an arbitrary number of meta-levels. For simplicity of presentation, we omit inheritance, cardinalities and integrity constraints in our formalization.

**Def. 1 (Deep model)** *A deep model is a tuple* $M = \langle p, C, S, R, src, tar, pot\rangle$*, where:*

- $p \in \mathbb{N}_0$ *is called the model potency, or* level.
- $C, S$ *and* $R$ *are disjoint sets of clabjects, slots and references, respectively.*
- $src\colon S \cup R \to C$ *is a function assigning slots and references to clabjects.*
- $tar\colon R \to C$ *is a function assigning the target clabject to references.*
- $pot\colon C \cup S \cup R \to \mathbb{N}_0$ *is a function assigning a potency to each element, s.t.:*
  1. $\forall e \in C \cup S \cup R \bullet pot(e) \leq p$
  2. $\forall s \in S \cup R \bullet pot(s) \leq pot(src(s))$
  3. $\forall r \in R \bullet pot(r) \leq pot(tar(r))$

In the previous definition, we assign a level $p$ to deep models. Elements in a deep model have a potency via function *pot*, which must satisfy three conditions: (1) the potency of an element should not be larger than the model level, (2) the potency of slots and references should not be larger than the one of their container clabject, and (3) the potency of references should not be larger than the one of the clabjects they point to.

Next, we define a general notion of mapping (a *morphism*) between deep models as a tuple of three (total) functions between the sets of clabjects, slots and references. Each morphism has a *depth* (an integer or 0) controlling the distance between the levels of the involved models. We use two particular types of mappings to represent the *type* relation between deep models at adjacent meta-levels (when the morphism depth is 1), and *extensions* of a deep model to add linguistic extensions (when the depth is 0).

**Def. 2 (D-morphism, type and extension)** *Given two deep models* $M_i = \langle p_i, C_i, S_i, R_i, src_i, tar_i, pot_i\rangle$ *for* $i = \{0, 1\}$*, a deep model morphism (D-morphism in short)* $m = \langle d, m_C, m_S, m_R\rangle\colon M_0 \to M_1$ *is a tuple made of a number* $d \in \mathbb{N}_0$ *called depth, and three functions* $m_C\colon C_0 \to C_1$*,* $m_S\colon S_0 \to S_1$ *and* $m_R\colon R_0 \to R_1$ *s.t.:*

1. $p_0 + d = p_1$
2. $\forall e \in X_0 \bullet pot_0(e) + d = pot_1(m_X(e))$ *(for* $X = \{C, S, R\}$*)*

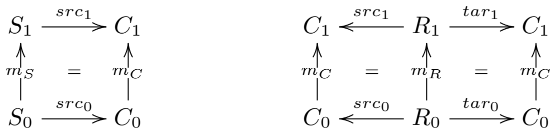

**Fig. 5.** Commutativity conditions for D-morphisms.

3. *Each function* $m_C, m_S, m_R$ *commutes with functions* $src_i$ *and* $tar_i$ *(see Fig. 5)*

*D-morphism* $tp = \langle d, tp_C, tp_S, tp_R\rangle \colon M_0 \to M_1$ *is called* type *if* $d = 1$*, and is called* indirect type *if* $d > 1$*.* $M_1$ *is called the (indirect) model type of* $M_0$*.*
*D-morphism* $ex = \langle d, ex_C, ex_S, ex_R\rangle \colon M_0 \to M_1$ *is called* level-preserving *if* $d = 0$*. A level-preserving D-morphism* $ex$ *is called* extension *if each* $ex_X$ *(for* $X = \{C, S, R\}$*) is an inclusion. An extension is called* identity *if each* $ex_X$ *is surjective.*

In the previous definition, condition 1 ensures that the D-morphism connects models of suitable levels, condition 2 checks that the potency decreases according to the depth of the D-morphism, and condition 3 ensures that the D-morphism is coherent with the source and target of slots and references (just like in standard graph morphisms [16]). We use total functions to represent the type, which ensures that each element in a deep model has a type. Linguistic extensions are not typed, but they are modelled as an extension D-morphism of a (typed) deep model into a larger model. This avoids resorting to partial functions to represent the type, which would complicate the formalization [38]. Identity extensions map isomorphic deep models. D-morphisms can be composed by composing the three mappings and adding their depths.

A multi-level model is made of a root deep model, and a sequence of instantiations and extensions. The length of this sequence is equal to the root model level. The extensions are allowed to be identity extensions.

**Def. 3 (Multi-level model)** *A multi-level model* $MLM = \langle M'_0, ML = \langle (M'_i \xleftarrow{tp_{i+1}} M_{i+1} \xrightarrow{ex_{i+1}} M'_{i+1})\rangle_{i=0..p'_0-1}\rangle$ *is made of a deep model* $M'_0$ *called the root and a sequence* $ML$ *(of length* $p'_0$*, the level of* $M'_0$*) of spans of D-morphisms, where the left D-morphism is a* type *and the right D-morphism a (possibly identity)* extension.

*Example.* Fig. 6 shows a multi-level model (an excerpt of the one in Fig. 3) according to Def. 3. Slots are represented as rounded nodes, instead of inside the owner clabject box. In Fig. 3, we do not show slots with potency bigger than 0 that are typed, like Design.duration at level 1, which is omitted. However, such instances do exist, and are explicitly shown in Fig. 6 (see slot duration'@1 in models $M_1$ and $M'_1$). If a model does not include linguistic extensions (like $M_2$), then we use the identity extension D-morphism. Finally, it would be possible to derive the (indirect) type of $M_2$ w.r.t. $M'_0$ by defining a construction akin to a pullback that yields the part of $M_2$ typed by $M_1$ [28].

## 4 Multi-level model product lines

In order to solve the challenges identified in Section 2.2, we extend deep models with closed variability options by borrowing concepts from product lines. We use feature models [24] to represent the allowed variability.

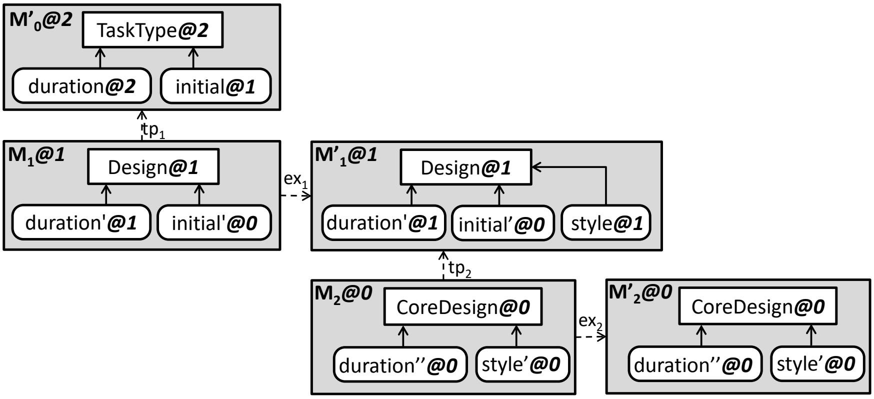

**Fig. 6.** Multi-level model example, according to Def. 3.

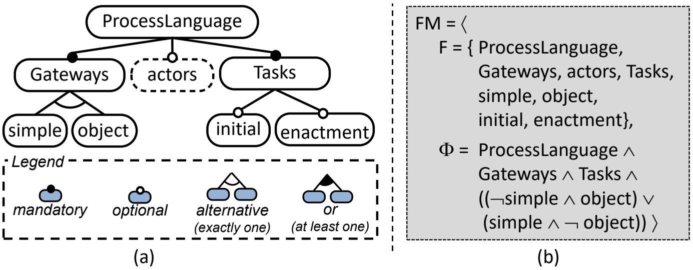

**Fig. 7.** Feature model for the example. (a) Feature diagram notation. (b) Using Def. 4.

**Def. 4 (Feature model)** *A feature model* $FM = \langle F, \Phi \rangle$ *is a tuple made of a set* $F$ *of features and a propositional formula* $\Phi$ *specifying the valid feature configurations.*

*Example.* Fig. 7 shows the feature model for the running example using both the feature diagram notation (a), and our definition (b). The feature model permits choosing if the process modelling language will have primitives to define actors (feature actors, cf. Fig. 4(b)), initial tasks and their enactment at level 0 (features initial and enactment, cf. Fig. 4(a)), as well as selecting whether gateways are to be represented either as references or objects (features simple and object, cf. Fig. 4(c)). The feature model includes the mandatory features ProcessLanguage, Gateways and Tasks as syntactic sugar to obtain a tree representation, but they are not needed in our formalization.

The selection of one option within the variability space offered by a feature model is done through a configuration. This assigns *true* to the selected features, and *false* to the discarded ones. To enhance flexibility of use, we also support partial configurations, where some features are not given any value. This will be used to allow deferring the resolution of some variability options to lower meta-levels.

**Def. 5 (Configuration)** *Given a feature model* $FM = \langle F, \Phi\rangle$, *a* configuration *of* $FM$ *is a tuple* $C = \langle F^+, F^-\rangle$ *made of two disjoint sets* $F^+ \subseteq F$ *and* $F^- \subseteq F$, *s.t.* $\Phi[F^+/true, F^-/false] \not\cong false$. $C$ *is* total *if* $F = F^+ \cup F^-$, *otherwise it is* partial.

In the previous definition, $F^+$ contains the selected features (i.e., given the value *true*), $F^-$ the discarded features (i.e., given the value *false*), and $F \setminus (F^+ \cup F^-)$ is the set of features whose value has not been set. A configuration must be compatible with the feature model formula, so the definition demands that the formula $\Phi$ once we substitute $F^+$ by *true* and $F^-$ by *false* is not false. If the configuration is total, then the condition entails that $\Phi$ must evaluate to true.

Next, we assign a level to feature models, and potencies to features, in order to restrict the level at which features can be assigned a value.

**Def. 6 (Deep feature model)** *A* deep feature model $DFM = \langle l, FM = \langle F, \Phi\rangle, pot\rangle$ *is made of a level* $l \in \mathbb{N}_0$, *a feature model* $FM$, *and a function* $pot\colon F \to \mathbb{N}_0$ *assigning a potency to each feature, s.t.* $\forall f \in F \bullet pot(f) \leq l$.

Next, we define a mapping between deep feature models, called F-morphism. Similar to D-morphisms (cf. Def. 2), F-morphisms have a depth which can be positive or 0. In addition, they include a configuration, and a mapping for the features excluded from the configuration. There are two special kinds of F-morphisms: one representing a type relationship between feature models (where the morphism depth is 1 and the configuration empty), and the other expressing a specialization relationship between two feature models via a total or partial configuration (where the morphism depth is 0).

**Def. 7 (F-morphism, type and specialization)** *Given two deep feature models* $DFM_i = \langle l_i, FM_i, pot_i\rangle$ *(for* $i = \{0, 1\}$*), a deep feature model morphism (F-morphism in short)* $m = \langle d, m_F, C\rangle\colon DFM_0 \to DFM_1$ *is made of:*

- *a depth* $d \in \mathbb{N}_0$ *s.t.* $l_0 + d = l_1$
- *an injective set morphism* $m_F\colon F_0 \to F_1$ *s.t.* $\forall f \in F_0 \bullet pot_0(f) + d = pot_1(m_F(f))$
- *a configuration* $C = \langle F_1^+, F_1^-\rangle$ *of* $FM_1$ *s.t.:*
  1. $m_F(F_0) = F_1 \setminus (F_1^+ \cup F_1^-)$
  2. $\Phi_1[F_1^+/true, F_1^-/false] \cong \Phi_0[F_0/m_F(F_0)]$

*F-morphism tp is a* type *morphism if* $d = 1$ *and* $C = \langle\emptyset, \emptyset\rangle$, *and it is an* indirect type *morphism if* $d > 1$ *and* $C = \langle\emptyset, \emptyset\rangle$. *F-morphism sp is a* specialization *if* $d = 0$.

The definition requires that the F-morphism depth fills the gap between the feature model levels, and between the potencies of the mapped features. $FM_0$ may have fewer features than $FM_1$, in case the configuration $C$ assigns a value to features of $FM_1$. In particular, the injectivity condition of $m_F$ and requiring $m_F(F_0) = F_1 \setminus (F_1^+ \cup F_1^-)$ ensures that only the features left undefined by $C$ are mapped from $FM_0$. Moreover, when the configuration $C$ assigns a value to some feature, we require that the formula $\Phi_1$, once we substitute the features in $C$ by their value *true* or *false*, be equivalent to $\Phi_0$, once we substitute the features in $F_0$ by their mapping in $F_1$. This corresponds to a (partial) evaluation of the formula $\Phi_1$ as a result of a feature model specialization.

As a remark, F-morphisms so defined are composable by adding their depths and making the union of the positive (resp. negative) features in the configurations.

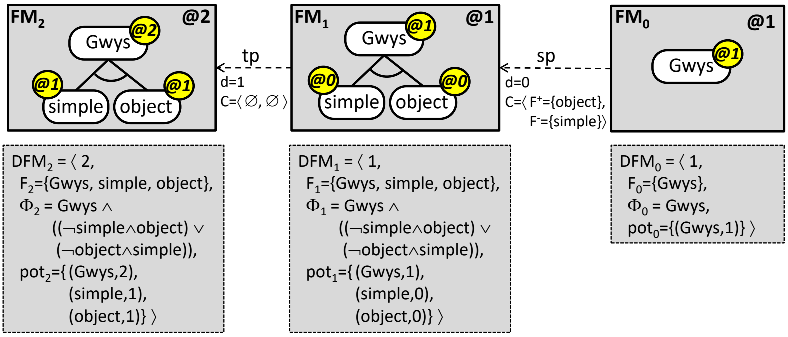

**Fig. 8.** Examples of F-morphisms.

*Example.* Fig. 8 shows two F-morphisms, with $tp$ a type and $sp$ a specialization. F-morphism $tp\colon FM_1 \to FM_2$ relates two deep feature models $FM_1$ and $FM_2$, where the level and potencies of $FM_1$ are one less than those in $FM_2$, and the formulae are the same modulo feature renaming. Specialization $sp\colon FM_0 \to FM_1$ has depth 0 and partial configuration $C = \langle F^+ = \{\mathsf{object}\}, F^- = \{\mathsf{simple}\}\rangle$. Hence, the levels and potencies are maintained, but the feature set $F_0$ is decreased by removing from $F_1$ the features that appear in $C$. According to condition 1 in Def. 7, $\{\mathsf{Gwys}\} = \{\mathsf{Gwys}, \mathsf{simple}, \mathsf{object}\} \setminus (\{\mathsf{simple}\} \cup \{\mathsf{object}\})$. According to condition 2 in the definition, the formula $\Phi_0$ is equivalent to replacing object by *true* and simple by *false* in $\Phi_1$. If we compose $sp$ with $tp$, the resulting F-morphism $tp \circ sp$ has depth 1 and configuration $C = \langle F^+ = \{\mathsf{object}\}, F^- = \{\mathsf{simple}\}\rangle$, which is neither a type nor a specialization.

Finally, we are ready to characterize deep model product lines (PLs) as a deep model, a deep feature model with the same level as the deep model, and a mapping of presence conditions (PCs) to deep model elements.

**Def. 8 (Deep model PL)** *A deep model PL* $DM = \langle M, DFM, \phi\rangle$ *is made of:*

- *A deep model* $M$ *and a deep feature model* $DFM$ *with the same level* ($p = l$)*.*
- *A function* $\phi\colon C \cup S \cup R \to \mathbb{B}(F)$ *mapping each element in* $M$ *to a (non-false) propositional formula over the features in* $F$*, called presence condition (PC), s.t.:*
  1. $\forall s \in S \cup R \bullet \phi(s) \implies \phi(src(s))$
  2. $\forall r \in R \bullet \phi(r) \implies \phi(tar(r))$
  3. $\forall e \in C \cup S \cup R,\ \forall v \in Var(\phi(e)) \bullet pot(v) \leq pot(e)$

Intuitively, given a configuration, we can derive a *product* (a deep model) of the PL by deleting the model elements whose PC evaluates to false. To avoid dangling references and slots, Def. 8 requires their PC not to be weaker than that of their owning clabject (condition 1), and the PC of references not to be weaker than the one of their target clabject (condition 2). In addition, the variability of an element must be resolved in a level that contains the element. To this aim, condition 3 ensures that the potency of the variables in the PC of an element is not higher than the element's potency (we use function Var to return all variables within a propositional formula).

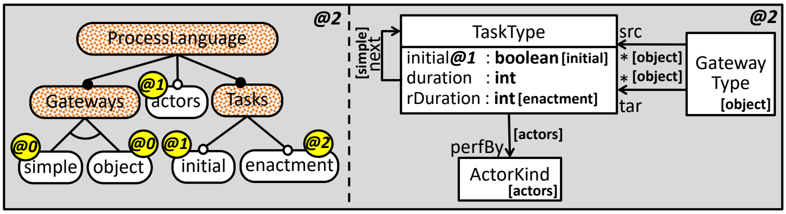

**Fig. 9.** Deep model PL example.

*Example.* Fig. 9 shows a deep model PL for process modelling languages. The left compartment shows the deep feature model, and the one to the right the deep model with its elements annotated with their PC between square brackets. If an element does not show a PC (like TaskType), then its PC is true. The deep model PL permits selecting between two alternative realizations for gateways, either as the reference next or the clabject GatewayType. This variability needs to be resolved before instantiating the language for a specific domain, as features simple and object have potency 0. The PL also offers the choice to add or not the primitive ActorKind to the language, but this decision can be taken before specializing the language or at level 1 to enable exploratory modelling. Finally, the PL allows selecting whether tasks can be initial and whether they hold enactment information. Feature initial in the feature model cannot have potency 2 because the feature is used in the PC of attribute TaskType.initial, which has potency 1. The feature model shows features ProcessLanguage, Gateways and Tasks in colour and without a potency; this is so as these features are mandatory (i.e., their value is *true* in any valid configuration), and while they enable a hierarchical representation of the feature model, the formalization of the example does not include them.

Next, we introduce mappings between deep model PLs (called PL-morphisms) as a tuple of morphisms between their constituent deep models and deep feature models. As in the previous cases, we are interested in type morphisms, linguistic extensions, and specializations of deep model PLs via a (partial) configuration.

**Def. 9 (PL-morphism, type, extension, specialization)** *Given two deep model PLs* $DM_i = \langle M_i, DFM_i, \phi_i \rangle$ *(for* $i = \{0, 1\}$*), a PL-morphism* $m = \langle m^D, m^F \rangle$ *is made of a D-morphism* $m^D : M_0 \to M_1$ *and an F-morphism* $m^F : DFM_0 \to DFM_1$ *with configuration* $C = \langle F^+, F^- \rangle$*, s.t.* $\forall e \in C_0 \cup S_0 \cup R_0 \bullet \phi_1(e)[F_1^+/true, F_1^-/false] \cong \phi_0(e)[F_0/m_F^F(F_0)]$.
*PL-morphism* $tp = \langle tp^D, tp^F \rangle$ *is a* type *if both* $tp^D$ *and* $tp^F$ *are types.*
*PL-morphism* $ex = \langle ex^D, id^F \rangle$ *is an* extension *if* $ex^D$ *is an extension and* $id^F$ *is an identity.*
*PL-morphism* $sp = \langle m^D, sp^F \rangle$ *is a* specialization *if* $sp^F$ *is a specialization and* $m^D$ *is injective, level-preserving, and the elements* $e \in C_1 \cup S_1 \cup R_1$ *s.t.* $\phi_1(e)[F_1^+/true, F_1^-/false] \ncong false$ *are in its co-domain.*

*Remark.* No condition on the equality of depths of $m^D$ and $m^F$ is required, since the levels of $M_0$ and $DFM_0$ are the same (and similar for the levels of $M_1$ and $DFM_1$).

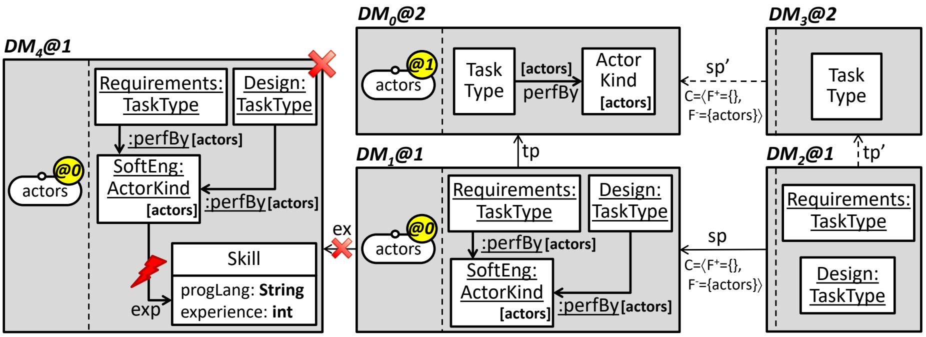

**Fig. 10.** Examples of PL-morphisms and deferred configuration.

The condition for PL-morphisms demands that the PCs in the deep model $M_0$ are modified according to the selection of features in configuration $C$ of $m^F$. In addition, in specialization PL-morphisms, $M_0$ should contain just the elements whose PC is not false after substituting the features in $F^+$ by *true*, and the ones in $F^-$ by *false*. Therefore, in case of a specialization, the definition requires that, when the configuration $C$ is considered, exactly the elements in $M_1$ whose PC is not false receive a mapping from $M_0$, while the mapping needs to be injective. Moreover, by Def. 8 of deep model PL, no element in $M_0$ can have a PC that is false.

Other kinds of PL-morphisms are possible, for example, adding features to a feature model in lower meta-levels to increase its variability. While this is an interesting possibility to increase language reuse, we leave its formalization to future work.

*Example.* Fig. 10 shows four valid PL-morphisms ($tp, tp', sp, sp'$) and an invalid one ($ex$). Both $tp$ and $tp'$ are types: they relate models at adjacent levels, where one is an instance of the other. Types always use the empty configuration $C = \langle \emptyset, \emptyset \rangle$ (cf. Def. 7), and therefore, a model element and its instances have the same PC (see, e.g., ActorKind and its instance SoftEng). Both $sp$ and $sp'$ are specialization PL-morphisms. This is so as they preserve level and potencies, and the deep models only contain elements with non-false PC. As the configuration $C$ of both PL-morphisms is total, the PC of the elements in $DM_3$ and $DM_2$ evaluates to true, and hence, these models do not have more closed variability options to configure (i.e., they are final products of the PL). The figure also shows an attempt to extend $DM_1$ by a linguistic extension made of the clabject Skill connected to SoftEng through reference exp. However, the result is not a valid deep model PL as the PC of SoftEng (*actors*) is stronger than the PC of exp (true). This could be solved by adding *actors* as PC of exp (and Skill).

When the configuration $C$ of a specialization PL-morphism $sp$ is total, $DM_0$ is a *product* of $DM_1$ with no variability, being equivalent to a deep model (cf. Def. 1). However, the question remains whether for any valid configuration $C$ of a deep model PL $DM$, we can find a deep model PL $DM'$ and a specialization PL-morphism $sp\colon DM' \to DM$ that uses $C$. This requires showing that any choice of $F^+$ and $F^-$ results in a valid deep model PL $DM'$ as given by Def. 8. Theorem 1 captures this result.

**Theorem 1 (Derivation through specialization morphisms).** *Given a deep model PL $DM = \langle M, DFM, \phi \rangle$ and any configuration $C$ of $DFM$, there is one deep model PL $DM'$ and a specialization morphism $sp\colon DM' \to DM$ with configuration $C$.*

*Proof.* In appendix.

Next, we look into the soundness of deferring the configuration of an element after it is instantiated. The question is whether, in any situation that allows configuring an element after its instantiation, we obtain the same result by resolving the element variability first and then instantiating. This result is important as, regardless of the order in which configurations and instantiation are performed, we can calculate the language that results of applying the configurations as the first step, by advancing the configuration steps over the instantiations.

The next theorem captures the fact that if we can instantiate and then configure, then we obtain the same result if we configure and then instantiate.

**Theorem 2 (Specialization can be advanced to instantiation).** *Given three deep model PLs $DM_i = \langle M_i, DFM_i, \phi_i \rangle$ (for $i = \{0, 1, 2\}$), a type PL-morphism $tp\colon DM_1 \to DM_0$ and a specialization PL-morphism $sp\colon DM_2 \to DM_1$, there is a unique deep model PL $DM_3$, a unique type PL-morphism $tp'\colon DM_2 \to DM_3$ and a unique specialization PL-morphism $sp'\colon DM_3 \to DM_0$ s.t. the diagram in Fig. 11 commutes.*

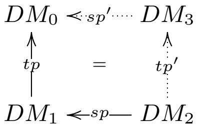

**Fig. 11.** Deferred configuration: specialization can be advanced to instantiation.

*Proof.* In appendix.

*Remark.* Note that the converse is not true in general, that is, instantiation cannot be advanced to specialization. The reason is that a type morphism is not allowed from features with potency 0, meaning that they must be configured first.

*Example.* Fig. 10 shows a deferred configuration. Deep model PL $DM_0$ is instantiated into $DM_1$, and then configured using $C = \langle F^+ = \{\}, F^- = \{actors\} \rangle$ to yield $DM_2$. Instead, we obtain the same result by first configuring $DM_0$ to yield $DM_3$, and then instantiating $DM_3$ into $DM_2$. Deep model PL $DM_3$ is relevant as it corresponds to the fully-configured language (i.e., with no variability) employed to build $DM_2$.

## 5 Tool support

We have implemented the notions presented so far atop METADEPTH [12]. This is a textual multi-level modelling tool which supports an arbitrary number of meta-levels and

```
1  @Variability(model="ProcessOptions")
2  Model ProcessModel@2 {
3    Node TaskType {
4      @Presence(condition="initial")
5      initial@1 : boolean = false;
6      duration : int;
7      @Presence(condition="enactment")
8      rDuration : int;
9      @Presence(condition="simple")
10     next : TaskType;
11     @Presence(condition="actors")
12     perfBy : ActorKind;
13   }
14
15   @Presence(condition="actors")
16   Node ActorKind;
17
18   @Presence(condition="object")
19   Node GatewayType {
20     src : TaskType[*];
21     tar : TaskType[*];
22   }
23 }
```

**Listing 1.** Deep model with PCs.

```
FeatureModel ProcessOptions@2 {
  ProcessLanguage : Gateways Tasks actors?@1;
  alt Gateways : simple@0 object@0;
  Tasks : initial?@1 enactment?@2;
}
```

**Listing 2.** Deep feature model.

```
config ProcessModel with { !simple }
```

**Listing 3.** Feature configuration.

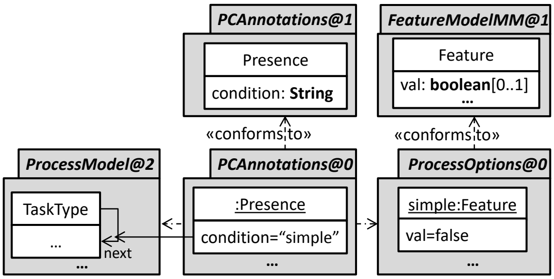

**Fig. 12.** Internal representation of deep model PL.

deep characterization through potency. It integrates the Epsilon family of languages for model management [33], which permits defining code generators and model transformations for multi-level models.

MetaDepth was used to define language families via multi-level modelling in [15], but it did not support the definition of closed sets of variability options by means of PLs. For this work, we have extended the tool to allow creating deep feature models and multi-level models with PCs, and specializing deep model PLs via configurations. The extended tool is available at `http://metadepth.org/pls`.

Listing 1 specifies the deep model in the right part of Fig. 9, using MetaDepth's syntax. First, line 1 states the name of the deep feature model (defined in Listing 2) associated to the deep model. Then, line 2 declares the deep model, named ProcessModel, with level 2. This contains three clabjects: TaskType (lines 3–13), ActorKind (lines 15–16) and GatewayType (lines 18–22). PCs are specified as annotations. This is possible as, similar to Java [10], MetaDepth permits defining annotation types by providing their syntax, parameters, and kind of elements they can annotate (i.e., models, clabjects or fields) [40]. This definition is a meta-model, and so, when annotations are parsed, they are transformed into an annotation model that refers to the annotated model. Regarding the PC of fields, for usability reasons, our implementation internally conjoins the PC of fields with the PC of their owner clabject. For example, the PC of reference GatewayType.src is *object* because the PC of GatewayType is *object*.

Listing 2 shows the MetaDepth definition of the deep feature model in Fig. 9. This conforms to a meta-model that we have created to represent deep feature models, and to which we have assigned a concrete syntax similar to the FAMILIAR tool [1]. Line 1 declares a feature model called ProcessOptions with level 2. Line 2 declares the root feature ProcessLanguage, and its children features Gateways, Tasks and actors. Children features can specify a potency after the "@" symbol, and be declared optional using the "?" symbol. Line 3 declares the children of Gateways, which are alternative as specified by the keyword alt. Line 4 declares the children of Tasks, which are optional.

Fig. 12 shows the internal representation of a deep model PL in METADEPTH. The PC annotations are automatically converted into an annotation model, which is also linked to the deep feature model (ProcessOptions).

Annotations in METADEPTH can attach actions to be triggered upon certain modelling events, like instantiation or value assignment. These actions are defined via a meta-object protocol (MOP) [26, 40]. This way, we have defined a MOP with actions for the PC annotations, to help instantiating deep model PLs. Specifically, when an element of a model with variability is instantiated (like ProcessModel in Listing 1), its PC is copied to the instance. Moreover, a constraint forbids instantiating a deep model PL if the associated deep feature model has features with potency 0.

Finally, we have created a command called config to specialize a deep model PL via a configuration (see Listing 3). When the command is applied, the PCs attached to model elements are evaluated (partially if the configuration is partial), and then removed if their value is *false*. The applied configuration (i.e., the boolean values assigned to the features) is stored in the deep feature model itself (cf. model ProcessOptions in Fig. 12). Overall, this simple example language already admits 16 total configurations, which can be succinctly represented as a PL, increasing its reuse possibilities.

## 6 Related work

Next, we review related research coming from language PLs; variability in multi-level modelling; and SPLs.

**Language PLs.** Some researchers have proposed increasing the reusability of modelling languages by incorporating SPL techniques. For example, in [47], DSL meta-models can be configured using a feature model. In [34], the authors propose featured model types: meta-models whose elements have PCs, and with operations that are offered depending of the chosen variant. In [20], meta-models can have variability, and their instantiability is analysed at the PL level. However, all these works only consider closed variability, while our work also supports open variability through instantiation.

**Variability in multi-level modelling.** A plethora of multi-level modelling approaches and tools have emerged recently, like DeepTelos [22], FMMLx [18], Melanee [3], MultEcore [29], MLT [17] and OMLM [21]. Some of them are based on deep characterization through potency [3, 18, 21, 29], while others rely on powertypes [17] or most-general instances [22]. None of them support variability based on feature models as we describe here. However, there have been some attempts to improve multi-level modelling with SPL techniques, which we describe next.

Reinhartz-Berger and collaborators [37] present a preliminary proposal to support the configuration of classes with optional attributes. It is based on a *kernel language*

which supports multiple meta-levels but not deep characterization. The proposal is incipient as it is neither formalized nor implemented. In [9], the authors analyse the limitations of feature models alone to describe a set of assets, and propose using multi-level models instead. As multi-level models have limitations to express variability – as described in Section 2.2 – we propose to combine feature models and multi-level models.

Nesic and collaborators [31] explore the use of MLT [17] to reverse engineer sets of related legacy assets into PLs. MLT is a multi-level modelling approach based on powertypes and first order logic. In their work, the authors represent variability concepts like PCs and product groups within MLT models. This embedding may result in complex models where elements can represent either variability concepts or domain concepts. Instead, we separate PCs and feature models to avoid cluttering the multi-level model. Our goal is to define highly reusable language families, for which we provide feature models to describe variability options, and offer the possibility to defer configurations; instead, the approach in [31] lacks an explicit representation of feature models. Finally, we provide both a theory and a working implementation.

Other formalizations of potency-based multi-level modelling exist, like [38]. That theory does not account for variability, but it could be extended with feature models, in a similar way as we do.

**SPLs.** Our deferred configurations can be seen as a particular case of *staged configurations* [11]. These permit selecting a member of the PL in stages, where each stage removes some choices. In our approach, the *potency* controls the level where the variability can be resolved. Staged configurations are also useful in software design reuse. In this setting, Kienzle and collaborators [27] propose Concern-Oriented Reuse, a paradigm where reusable modules (called *concerns*) define variability interfaces as feature models. The variability of a reused concern can be resolved partially, in which case, the undefined features are re-exposed in the interface of the resulting concern. We also support deferring the variability resolution, but composing deep model PLs is future work.

Taentzer and collaborators [45] formalized model-based SPLs using category theory. Different from ours, their formalization does not capture typing (it is within a single meta-level), while their morphisms can expand the feature model but cannot be used to model partial configurations. Borba and collaborators [8] have studied PL refinements to add new products maintaining the behaviour of existing ones. In our case, we do not increase variability, but it would be interesting to consider mechanisms to do so combined with instantiation.

To cope with large variability spaces, partitioning techniques can be applied to feature models to yield so-called multi-level feature models [11, 36]. However, the term multi-level does not refer to multiple levels of classification (as in our case), but to multiple partitions of a feature model.

Other modelling notations support variability. For example, Clafer [23] is an approach that unifies feature and class modelling. It supports both class and (partial) object models, feature models, (partial) configurations and logic constraints. However, it does not support multi-level modelling or deep characterization. Similar to delta-oriented programming [42], $\Delta$-modelling [41] permits defining a set of products as a core model plus a set of modification deltas to the core model according to given application conditions. The approach has been combined with MDE, showing that model configuration and refinement (e.g., a component being refined by a set of classes) commute. This is in line with our Theorem 2, but we are interested in instantiation (instead of refinement), and need to incorporate potency for deep characterization. Hence, in our case, instantiation and specialization (configuration) do not commute, but the latter can be advanced to former.

In the programming world, Batory [7, 44] proposes mixin layers, a composition mechanism to add features to sets of base classes (so called two-level designs). Higher-level designs can be obtained by applying the same techniques. In [7], these higher-level designs are called multi-level models. Again, the use of the term multi-level is different from ours, which refers to models related by classification relations.

Overall, our proposal is the first one adding variability to multi-level models with support for deep characterization.

## 7 Conclusions and future work

In this paper, we have proposed a new notion of multi-level model PL to improve current reuse techniques for modelling languages. This is so as it permits both *open* variability (by successive instantiations leading to language refinements for specific domains), and *closed* variability (by selecting among a set of variants). We have presented a theory, with results ensuring the proper interleave of instantiation and configuration steps. The ideas have implemented on top of the multi-level modelling tool METADEPTH.

In the future, we plan to provide a categorical formalization of the theory which brings operations like intersection via common parts (pullbacks) and merging (pushouts) of deep model PLs. We also want to offer the possibility of extending a deep model PL with new features (i.e., extra variability) and move this variability to the top model whenever possible. We would like to develop analysis techniques for multi-level model PLs, e.g., to check instantiability properties in the line of [20]. Finally, our goal is to make multi-level model PLs ready for MDE. This would entail the ability to define MDE services like transformations and code generators on multi-level model PLs. Technically, our plan is to use the Epsilon languages supported by METADEPTH, and follow ideas from existing works on PLs of transformations [13], and transformation of PLs [39].

**Acknowledgments.** Work funded by the Spanish Ministry of Science (project MASSIVE, RTI2018-095255-B-I00) and the R&D programme of Madrid (project FORTE, P2018/TCS-4314). We thank the anonymous referees for their useful comments.

## Appendix

**Proof of Theorem 1**: Given a deep model PL $DM$ and a configuration $C = \langle F^+, F^- \rangle$, we build $DM' = \langle M', DFM', \phi' \rangle$ as follows:

- $M'$ has the same level as $M$, and contains the elements $e$ of $M$ s.t. $\phi(e)[F^+/true, F^-/\,false] \not\equiv false$. Functions $src'$, $tar'$ and $pot'$ are restrictions of $src$, $tar$ and $pot$ to the elements in $M'$.

- $DFM' = \langle l, FM' = \langle F', \Phi' \rangle, pot' \rangle$, where $F' = F \backslash (F^+ \cup F^-)$, $\Phi' = \Phi[F^+/true, F^-/false]$, and $pot'$ is the restriction of $pot$ to $F'$.
- $\forall e \in C' \cup S' \cup R' \bullet \phi'(e) = \phi(e)[F^+/true, F^-/false]$.

Now we show that $M'$ is a valid deep model according to Def. 1:

- To check that $src'$ is well formed, we show that $\forall s \in S' \cup R'$, $src(s')$ is defined. By condition 1 in Def. 8, $\phi(s) \implies \phi(src'(s))$. This precludes the source of any $s \in S' \cup R'$ to be absent from $C'$, since if $\phi(src'(s))[F^+/true, F^-/false] = false$, then $\phi'(s)[F^+/true, F^-/false] = false$.
- The well-formedness of $tar'$ is shown like in the previous case.
- Function $pot'$ satisfies conditions 1–3 of Def. 1, since $pot$ satisfies them, and $pot'$ is just a restriction of $pot$.

Now we show that $DM'$ is a valid deep model PL according to Def. 8:

- $M'$ and $DFM'$ have the same level ($l$).
- The three conditions over $\phi'$ and $pot'$ hold, since they hold for $\phi$ and $pot$.

Finally, we build a specialization PL-morphism $sp = \langle m^M, sp^F \rangle : DM' \to DM$ as follows:

- $m^M = \langle 0, inc^M_C, inc^M_S, inc^M_R \rangle$, where $X' \xhookrightarrow{inc^M_X} X$ (for $X = \{C, S, R\}$) are inclusion set morphisms,
- $sp^M = \langle 0, inc^F, C \rangle$, where $F' \xhookrightarrow{inc^F} F$ is an inclusion morphism.

We need to show that: (i) $m_F(F') = F' = F \backslash (F^+ \cup F^-)$, which holds since $F'$ was defined above as $F \backslash (F^+ \cup F^-)$; and (ii) $\Phi[F^+/true, F^-/false] \equiv \Phi'[F'/inc_F(F')]$, which holds since $\Phi'$ was defined above as $\Phi[F^+/true, F^-/false]$. □

**Proof of Theorem 2**: Let $C = \langle F^+, F^- \rangle$ be the configuration of the specialization PL-morphism $sp \colon DM_2 \to DM_1$. From $DM_0$ and $C$, we construct a deep model $DM_3$ and a specialization PL-morphism $sp' \colon DM_3 \to DM_0$ as described in the proof of Theorem 1. Then, we build a type PL-morphism $tp' = \langle tp'^D, tp'^F \rangle : DM_2 \to DM_3$ as follows:

- $tp'^D = \langle 1, tp^D_C|_{C_2}, tp^D_S|_{S_2}, tp^D_R|_{R_2} \rangle$, with $tp^D_X|_{X_2}$ the restriction of $tp^D_X$ to set $X_2$ in $DM_2$ (for $X = \{C, S, R\}$).
- $tp'^F = \langle 1, tp^F_F|_{F_2}, C \rangle$ with $tp^F_F|_{F_2}$ the restriction of $tp^F_F$ to set $F_2$.

D-morphism $tp'^D$ is well defined because $\forall c \in C_2, \exists c' \in C_3$ s.t. $tp^D_C(sp^D_C(c)) = sp'^D_C(c')$. This is so as $\phi_1(sp^D_C(e))[F^+/true, F^-/false] \not\equiv false$ due to Def. 9 of specialization PL-morphism. And now, since the configuration of $tp$ is empty, we have $\phi_0(tp^D_C(sp^D_C(e))[F^+/true, F^-/false] \not\equiv false$. This means that, according to Def. 9, this element is in the co-domain of $sp'^D_C$, and is assigned to $c$ by $tp'^D_C$. The same reasoning applies to sets $S_2$ and $F_2$. Function $tp^F_F|_{F_2}$ is also well formed, since the same configuration $C$ was used to derive $DM_2$ and $DM_3$.

This reasoning also shows that $tp \circ sp = sp' \circ tp'$, as Theorem 2 demands. □
